# Do mosquitoes transmit the avian malaria-like parasite *Haemoproteus*? An experimental test of vector competence using mosquito saliva

**DOI:** 10.1186/s13071-016-1903-9

**Published:** 2016-11-28

**Authors:** Rafael Gutiérrez-López, Josué Martínez-de la Puente, Laura Gangoso, Jiayue Yan, Ramón C. Soriguer, Jordi Figuerola

**Affiliations:** 1 Department of Wetland Ecology, Estación Biológica de Doñana (EBD-CSIC), Avda. Américo Vespucio s/n,, E-41092 Sevilla, Spain; 2 CIBER Epidemiología y Salud Pública (CIBERESP), Sevilla, Spain

**Keywords:** *Culex pipiens*, Haemosporidians, Parasite transmission, *Plasmodium*, Vector-borne diseases

## Abstract

**Background:**

The life-cycle of many vector-borne pathogens includes an asexual replication phase in the vertebrate host and sexual reproduction in the insect vector. However, as only a small array of parasites can successfully develop infective phases inside an insect, few insect species are competent vectors for these pathogens. Molecular approaches have identified the potential insect vectors of blood parasites under natural conditions. However, the effectiveness of this methodology for verifying mosquito competence in the transmission of avian malaria parasites and related haemosporidians is still under debate. This is mainly because positive amplifications of parasite DNA in mosquitoes can be obtained not only from sporozoites, the infective phase of the malaria parasites that migrate to salivary glands, but also from different non-infective parasite forms in the body of the vector. Here, we assessed the vectorial capacity of the common mosquito *Culex pipiens* in the transmission of two parasite genera.

**Methods:**

A total of 1,560 mosquitoes were allowed to feed on five house sparrows *Passer domesticus* naturally infected by *Haemoproteus* or co-infected by *Haemoproteus/Plasmodium*. A saliva sample of the mosquitoes that survived after 13 days post-exposure was taken to determine the presence of parasite DNA by PCR.

**Results:**

Overall, 31.2% mosquito’s head-thorax and 5.8% saliva samples analysed showed positive amplifications for avian malaria parasites. In contrast to *Haemoproteus* DNA, which was not found in either the body parts or the saliva, *Plasmodium* DNA was detected in both the head-thorax and the saliva of mosquitoes. Parasites isolated from mosquitoes feeding on the same bird corresponded to the same *Plasmodium* lineage.

**Conclusions:**

Our experiment provides good evidence for the competence of *Cx. pipiens* in the transmission of *Plasmodium* but not of *Haemoproteus*. Molecular analyses of saliva are an effective method for testing the vector competence of mosquitoes and other insects in the transmission of vector-borne pathogens.

## Background

The avian malaria parasite *Plasmodium* and the malaria-like parasites of the genus *Haemoproteus* are pathogens that infect birds worldwide and cause infectious diseases that affect birds’ fitness [1, 2]. These parasites reproduce asexually in birds but are obliged to complete their sexual and sporogonic phases in their insect vectors before being successfully transmitted to a new vertebrate host. Mosquitoes (Diptera: Culicidae), especially those of the genus *Culex*, are the main vectors of avian *Plasmodium*; biting midges *Culicoides* (Diptera: Ceratopogonidae) and louse flies (Diptera: Hippoboscidae), on the other hand, transmit *Haemoproteus* (subgenera *Parahaemoproteus* and *Haemoproteus*) parasites, respectively [3, 4]. In mosquitoes, after the development of the ookinetes, parasites penetrate insects’ mid-gut walls and produce oocysts. These oocysts then divide to produce the sporozoites, the infective form of the malaria parasites, which migrate to the salivary glands of the mosquitoes. Sporozoites are thus transmitted by mosquito bites into the bloodstream of a new host [4].

Since the seminal paper by Bensch et al. [5], a number of different molecular approaches have been developed to study interactions between parasites and birds [6, 7]. These molecular methods are also a valuable tool for identifying the potential insect vectors of blood parasites under natural conditions [8, 9]. However, an intense debate exists regarding the reliability of molecular approaches in the study of vector competence [10, 11]. This controversy arises from the fact that positive amplification of parasite DNA can be obtained from insects due to the presence of non-infective forms of the parasite, which are unable to complete their multiplicative cycle. For instance, *Haemoproteus* DNA has been isolated from both *Culicoides* [12, 13] and several mosquito species, including *Culex pipiens,* which have completely digested blood meals [14–17]. All this evidence suggests that mosquitoes (and not only *Culicoides*) could be involved in the transmission of this parasite genus. Therefore, further studies are still required to determine the degree to which mosquitoes are competent in the transmission of *Haemoproteus* parasites.

We conducted an experimental study to determine, to our knowledge for the first time, the competence of *Cx. pipiens* mosquitoes in the transmission of avian malaria-like parasites of the genus *Haemoproteus. Culex pipiens* is a widely distributed mosquito species involved in the transmission of a number of vector-borne pathogens [18]. It is believed to be one of the main vectors of avian malaria parasites, and over 50 different genetic lineages have been detected in this mosquito species using molecular methods [14, 19]. To assess vector competence, mosquitoes were allowed to feed on wild birds naturally infected by *Haemoproteus* and birds co-infected by *Haemoproteus* and *Plasmodium* (individuals suffering co-infections are commonly found in the wild) [20–22]. After allowing the parasite to develop in the mosquito, we used molecular tools (PCR) to detect the presence of parasite DNA in the head-thorax (where the salivary glands are located) and saliva of mosquitoes. The detection of pathogens in mosquito saliva is frequently used in studies of the vector competence of pathogens such as West Nile virus [23] and Chikungunya virus [24] but, to the best of our knowledge, has never previously been employed to determine the vector competence of mosquitoes for avian malaria and malaria-like parasites.

## Methods

### Mosquito collection and rearing

*Culex pipiens* larvae were collected in La Cañada de los Pájaros, a natural reserve near Seville, Spain (6°14′W, 36°57′N). This area lies beyond the main wetlands of the Doñana National Park and consists of a freshwater lake (*c*.5 ha) surrounded by paddy fields. Larvae were transferred to the laboratory and kept in plastic trays with fresh water and fed *ad libitum* (Mikrozell 20 ml/22 g; Dohse Aquaristik GmbH & Co. KG, D-53501, Gelsdorf, Germany). Larvae and adult mosquitoes were maintained at constant conditions, 28 °C, 65–70% relative humidity (RH) and 12:12 light: dark cycle. After metamorphosis, adult mosquitoes were immediately placed in insect cages (BugDorm-43030F, 32.5 × 32.5 × 32.5 cm) and fed *ad libitum* with 1% sugar solution. Five to seven days later, adults were anesthetised with ether [25] and observed under a stereomicroscope (Nikon SMZ645) to determine their sex and confirm the species, following Schaffner et al. [26] and Becker et al. [27]. The sugar solution was replaced with water 24 h prior to each experiment (see below) and completely removed from cages 12 h before experiments began. The experiments were conducted using 13–22-day-old female *Cx. pipien*s.

### Bird trapping and sampling

Five juvenile (yearlings) house sparrows *Passer domesticus* were captured using mist nets on 15 July 2014 in Huelva province and subsequently ringed with numbered metal rings. To determine their haemosporidian infection status, a blood sample (0.2 ml) was taken from the jugular vein of each bird using sterile syringes and was then immediately transferred to non-heparinized Eppendorf tubes. Birds were transported to the Unit of Animal Experimentation at the Estación Biológica de Doñana (EBD-CSIC) and kept indoors in birdcages (58.5 × 25 × 36 cm) in a vector-free room under controlled conditions (23 ± 1 °C, 40–50% RH and 12:12 light: dark cycle). Birds were fed *ad libitum* with a standard mixed diet for seed-eating and insectivorous birds (KIKI; GZM S.L., Alicante, Spain). Three days after the last exposure to mosquitoes, birds were blood sampled again (0.2 ml; final blood samples) in the same way as above to detect any infections by blood parasites that could have not developed when initially sampled. Samples were not taken either immediately before or during the mosquito exposure period due to the stress caused by mosquito bites. Immediately after sampling, a drop of blood was smeared, air-dried, fixed in absolute methanol and stained with Giemsa for 45 min [28]. A total of 4,000–10,000 erythrocytes from each blood smear were scanned at high magnification (×1000) and the intensity of infection by *Haemoproteus*/*Plasmodium* parasites was estimated as the percentage of parasite cells per 100 erythrocytes. At the end of the experiment, birds were released at the capture site 23 days after being captured.

### Experimental procedure

Eleven days after capture, each bird was placed in a birdcage (38.5 × 25.5 × 26 cm) inside an insect tent (BugDorm-2120, 60 × 60 × 60 cm). Over four non-consecutive nights, each bird was introduced into an independent tent and exposed to 50 (first night), 57 (second night), 105 (third night) and 100 (fourth night) unfed *Cx. pipiens* females, summarizing a total of 312 mosquitoes per bird. The number of mosquitoes used each night varied according to the availability of unfed 13–22 days old mosquitoes. Birds were exposed to mosquito bites overnight (from 8:00 pm to 8:00 am). After exposure, mosquitoes with a recent blood meal in the abdomen were immediately separated and placed in unzipped insect cages (BugDorm-43030F 32.5 × 32.5 × 32.5 cm) and maintained under standard conditions (28 °C, 65–70% RH and 12:12 light: dark cycle). These mosquitoes had *ad libitum* access to 1% sugar solution during the following 13 days to allow parasite development.

### Sampling of mosquito saliva

Those mosquitoes that survived until 13 days post-exposure (dpe) were anesthetised with ether [25]. Mosquitoes’ legs and wings were removed with a sterile forceps. The mosquito proboscis was introduced into a 1 μl disposable capillary (Einmal-Kapillarpipetten, Hirschmann® Laborgeäte, Germany) filled with 1 μl of fetal bovine serum [29]. Then, 1 μl of 2% pilocarpine (Novartis 2012, Alcon Cusí S.A. Barcelona, Spain) was applied to the mosquito thorax to stimulate salivation [30]. After 45 min, the medium containing the saliva was placed in 1.5 ml Eppendorf tubes with 10 μl of MQ water and stored at −80 °C. Mosquitoes were kept in individual tubes at -80 °C until further molecular analysis. The head-thorax of eight mosquitoes and two saliva samples were not analysed due to logistical problems.

### Molecular detection and identification of blood parasites

DNA was isolated from birds’ blood samples (both the initial and final samples) and from the head-thorax of mosquitoes using a semi-automatic procedure (MAXWELL^®^ 16 LEV Blood DNA Kit) [31]. The Qiagen DNeasy^®^ Kit Tissue and Blood (Qiagen, Hilden, Germany) was used to isolate the DNA from saliva samples. A 478 bp fragment (excluding primers) of the mitochondrial cytochrome b gene of *Haemoproteus*/*Plasmodium* parasites was amplified following Hellgren et al. [6]. This procedure is based on a first PCR using primers HaemNFI (5′-CAT ATA TTA AGA GAA ITA TGG AG-3′) and HaemNR3 (5′-ATA GAA AGA TAA GAA ATA CCA TTC-3′), followed by a nested PCR using primers HaemF (5′-ATG GTG CTT TCG ATA TAT GCA TG-3′) and HaemR2 (5′-GCA TTA TCT GGA TGT GAT AAT GGT-3′). This procedure is able to detect parasite DNA in infections equivalent to less than one gametocyte per 10,000 erythrocytes in blood smears [6]. The presence of amplicons was verified in 1.8% agarose gels. Positive amplifications were sequenced in both directions using the BigDye technology (Applied Biosystems) or with the Macrogen sequencing service (Macrogen Inc., Amsterdam, The Netherlands). Sequences were edited using the software Sequencher™ v 4.9 (Gene Codes Corp. © 1991–2009, Ann Arbor, MI 48108, USA) and assigned to parasite lineages/morphospecies after comparison with the GenBank (National Center for Biotechnology Information) and Malavi [19] databases.

## Results

**Fig. 1:**
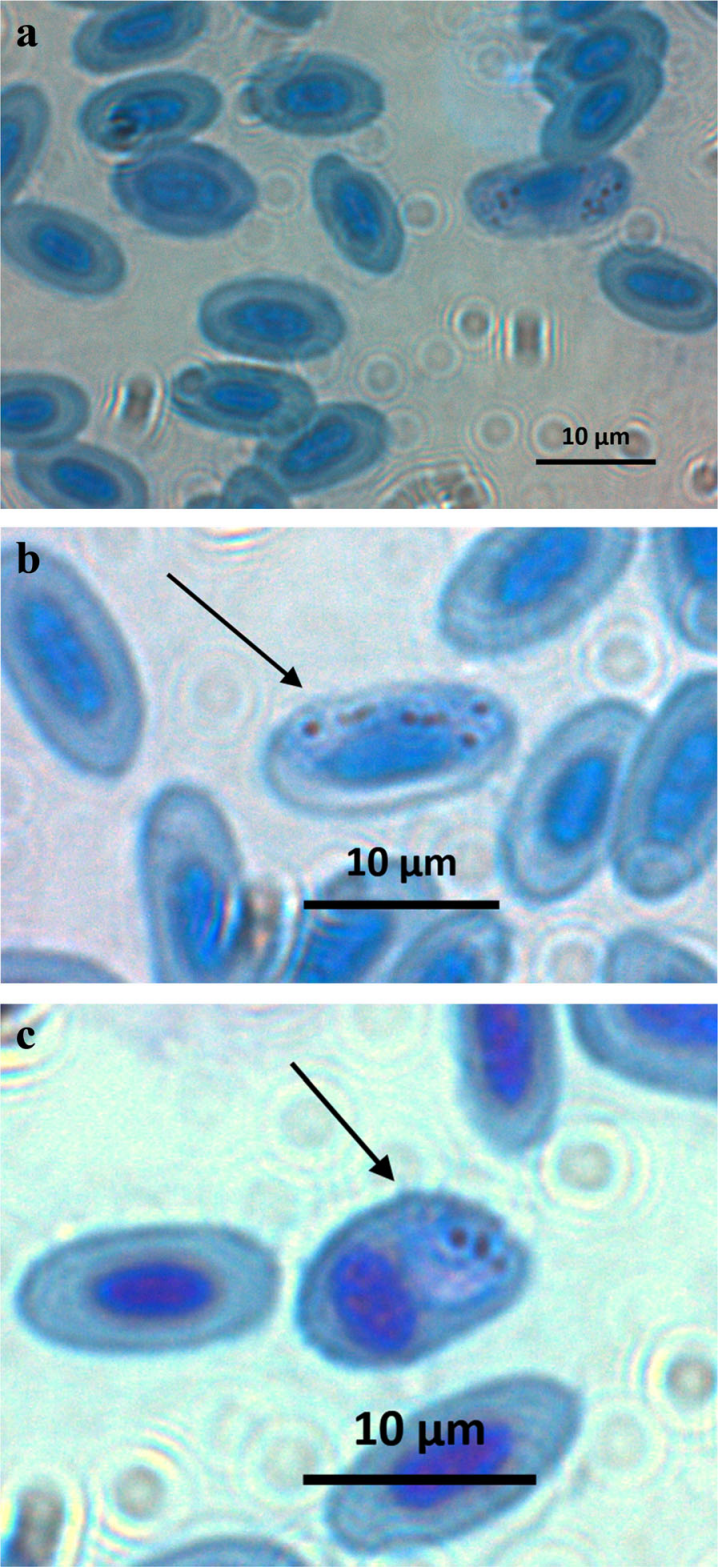
Blood parasites found in house sparrows (**a**) with details of *Haemoproteus passeris* (lineage padom05) (**b**) and *Plasmodium sp*. lineage padom01 (**c**). *Arrows* indicate the parasite cell

The five birds included in the study showed positive amplifications of blood parasites and there was no difference between initial and final samples. The parasite sequences isolated from all five birds had a 100% overlap with lineage *Haemoproteus* PADOM05 (corresponding to *H. passeris*). No evidence of double peaks in the chromatograms was found. The examination of blood smears revealed the presence of both *Haemoproteus* and *Plasmodium* parasites in two birds (house sparrows 4 and 5), only *Haemoproteus* in two other birds (house sparrows 2 and 3), and a total absence of parasites in one bird (house sparrow 1) (Fig. 1, Table 1).

**Table 1:** Infection status of birds included in this study and number of engorged and analyzed *Culex pipiens* mosquitoes

	Infection status (PCR)	Intensity of infection and morphological identification of parasites (blood smear)	Engorged mosquitoes	Alive mosquitoes after 13 days	Head-thorax positive/analysed	Saliva positive/analysed
House sparrow 1	*Haemoproteus*	*Haemoproteus* (0%)	9 (2.9%)	9 (100%)	0/9	0/9
House sparrow 2	*Haemoproteus*	*Haemoproteus* (0.4%)	39 (12.5%)	35 (89.7%)	0/34	0/34
House sparrow 3	*Haemoproteus*	*Haemoproteus* (0.2%)	42 (13.5%)	36 (85.7%)	1/36	0/36
House sparrow 4	*Haemoproteus*	*Haemoproteus* (0.5%)/*Plasmodium* (0.2%)	39 (12.5%)	33 (84.6%)	23/26	7/26
House sparrow 5	*Haemoproteus*	*Haemoproteus* (1.3%)/*Plasmodium* (0.3%)	45 (14.4%)	36 (80%)	20/36	1/34

Overall, 174 of 1560 (11.2%) mosquitoes used in this study fed on birds’ blood, 149 of them survived until 13 dpe. A total of 141 head-thorax and 139 saliva samples were molecularly analysed, of which 44 and 8 samples, respectively, were positive to parasite DNA (Table 1). All the saliva samples showing positive amplifications corresponded to mosquitoes with head-thorax that were also positive for parasite DNA. The parasite lineages isolated from the head-thorax and saliva of the mosquitoes that fed on the two co-infected birds, as revealed by the blood smears, corresponded to *Plasmodium* lineages. These lineages were identified as SGS1 (= Rinshi-1, corresponding to *Plasmodium relictum*) and PADOM01. We were unable to detect *Plasmodium* in the blood smear of one bird (identified as house sparrow 3, Table 1), probably due to a very low-intensity of infection, but did manage to isolate the *P. relictum* lineage GRW11 (= Rinshi-7) in the head-thorax of one of the 36 mosquitoes that fed on this bird (Table 1). Parasites isolated from mosquitoes feeding on the same individual corresponded to the same *Plasmodium* lineage. *Haemoproteus* was not found in either the head-thorax or in the saliva of any of the mosquitoes analysed.

## Discussion

Studies of host-parasite co-evolution in the context of avian malaria mainly focus on the interactions between parasites and their vertebrate hosts [32–34] but tend to ignore the role of invertebrate vectors. The development of avian blood parasites in mosquitoes is the outcome of a complex evolutionary ‘arms race’ too, in which the probability of encounter with mosquitoes and their compatibility are important obstacles for successful infection and the proper development of the parasites [35, 36]. Although *Cx. pipiens* females frequently feed on mammals, birds are their main blood-feeding source [18, 37, 38], a preference that may increase their contact rate with *Haemoproteus*. Nevertheless, our results suggest that mosquitoes actually may represent an obstacle to the successful development of the life-cycle of species in this parasite genus [36].

Here, we provide evidence of the effectiveness of mosquito saliva as a novel way of testing the vectorial competence of mosquitoes in the transmission of avian malaria and malaria-like parasites. This method has been commonly used in studies of the vector competence of mosquitoes in the transmission of a number of viruses that are of public health concern [39–42] as well as to detect proteins of *Plasmodium bergehi* sporozoites in the saliva of *Anopheles stephensi* [43]. However, to our knowledge, this approach has never been used in studies of mosquito-avian malaria interactions. Despite being time-consuming (it is possible to obtain the saliva of about 15 mosquitoes/h), this method is an excellent complementary procedure to the frequently used salivary gland dissection employed in studies on vector competence. By using this approach, it is possible to obtain parasite sporozoites while reducing/removing the presence of tissues derived from the salivary glands present in the sample. This could be of special relevance in studies on *Plasmodium* genotyping where the quantity of parasite DNA in relation to host DNA is an important limitation [44]. Moreover, mosquito saliva could be used in transcriptomic studies of the infective forms of avian malaria parasites and/or to study the parasite load inoculated by mosquitoes [45].

The lineages SGS1 (*P. relictum*) and PADOM01 were amplified in the saliva of mosquitoes at 13 dpe. However, a high percentage of mosquitoes with positive DNA amplifications in the head-thorax (81.8%) did not show positive *Plasmodium* DNA amplifications in saliva at 13 dpe. A recent study found that 13.3% of infected *Cx. pipiens* had *Plasmodium* sporozoites in their salivary glands [46], indicating that these parasites develop sporozoites in only a small percentage of infected mosquitoes. The absence of sporozoites in salivary glands could be explained by the fact that the parasite does not have enough time to complete its development until this phase. Thus, extracting saliva after 13 dpe could have increased the number of positive amplifications in our samples. However, some studies have found *Plasmodium* sporozoites in the salivary glands of mosquitoes from just 7 dpe [4, 47], although Kazlauskienė et al. [48] were unable to isolate sporozoites until 14 dpe in salivary glands (yet mosquitoes at 13 dpe were not analysed). The differences found between studies could be due to the use of different mosquito species, a differential mosquito microbiota, parasite strains, or environmental temperatures, which may greatly affect the ability of parasites to complete sporogony [4, 49, 50]. Unlike *Plasmodium*, the possibility that *Haemoproteus* had not have enough time to develop sporozoites is poorly supported. Previous studies using direct observational (microscope) and molecular (PCR) techniques found intermediate stages (i.e. ookinetes and oocysts) of *Haemoproteus* parasites in the head, thorax and/or abdomen of *Ochlerotatus cantans* mosquitoes from 4–6 dpe onwards, but presence of sporozoites was not recorded [51, 52]. By contrast, we found no evidence of *Haemoproteus* DNA in the head-thorax of the mosquitoes analysed. In addition, in their known *Culicoides* vectors, *Haemoproteus* sporozoites are also present in salivary glands at 5 dpe [53]. Therefore, our results support the inability of *Haemoproteus* lineage PADOM05 to complete its life-cycle in *Cx. pipiens.*

Molecular approaches allowing the identification of the parasite lineages harboured by insect vectors provide valuable information on the potential transmission networks of avian pathogens [12, 14, 16, 17]. Such tools enable a huge number of individuals (e.g. thousands of mosquitoes) to be handled, which is often necessary for detecting positive amplifications due to the low infection prevalence that is typical in mosquitoes trapped in the wild [15, 54, 55]. However, results from these studies should be interpreted with caution when attempting to identify the true vectors of avian pathogens, this is especially true when pathogen DNA is isolated from an unexpected vector, and highlights the necessity to conduct further experimental studies of vectorial competence [10]. Although different approaches including cloning and the development of specific primers have been employed to identify parasite lineages in co-infected birds [7, 56, 57], our results show the importance of combining the molecular detection of blood parasites with the analysis of blood smears when aimed at identifying potential co-infections in birds [58].

## Conclusions

The results from this study suggest that *Cx. pipiens* is unable to transmit *Haemoproteus* parasites. This study also highlights the value of targeting mosquito saliva as a means of assessing the competence of potential mosquito vectors in the transmission of avian *Plasmodium* lineages.

## Abbreviations

dpe: Days post-exposure
